# Restoration and the City: The Role of Public Urban Squares

**DOI:** 10.3389/fpsyg.2017.02093

**Published:** 2017-12-07

**Authors:** César San Juan, Mikel Subiza-Pérez, Laura Vozmediano

**Affiliations:** Social Psychology and Methodology of Behavioral Sciences, Faculty of Psychology, University of the Basque Country, San Sebastian, Spain

**Keywords:** attention restoration theory, stress recovery theory, field study, urban restoration, urban plaza

## Abstract

Over recent decades, the study of psychological restoration has attracted a considerable amount of interest within and without the boundaries of environmental psychology, with most of the work focused on analyzing restoration in natural contexts. However, little attention has been paid to the (possible) restorative potential of urban settings, as they have usually been expected not to be restorative and to present some elements that might imply negative health outcomes in the short and long term. In this field study, our aim was to evaluate restoration in urban squares. To this end, we measured participants' attentional and affective states both before and after spending half an hour in an urban square. A sample of 46 subjects contemplated and walked through one of the two selected squares that differed in restorative potential (PRS). Analyses revealed a statistically significant increase in cognitive performance and a decrease in negative affect in both squares. They also showed that participants reported greater stress recovery rates in one of the settings. These results support the idea that cities can be potentially restorative and justify the relevance of a research area focused on the urban designs, which may offer psychological benefits to urban citizens.

## Introduction

The potential for reducing negative psychological states or increasing positive ones is a well-established value present in natural landscapes, known as restorative potential. The possible restorative potential of urban landscapes has been less widely studied, since cities have been considered by authors such as Simmel (1984) and Milgram (1970) as being stressful and over-demanding contexts. Even when the urban environment has been studied, attention was focused on green areas within the city (e.g., urban parks, forests, and university campuses). Consequently, our knowledge of the potential role that other public urban places may play is limited, although some evidence supports the idea that urban landscapes may also be restorative. The aim of this paper is to expand existing evidence by assessing the psychological effects of spending time in a specific type of the urban landscape: public squares.

### The psychology of restoration: aesthetic experience and well-being

Landscape preferences have become a relevant field of research for several disciplines within the social sciences, particularly environmental psychology. The lack of “objective” criteria for establishing the quality of a landscape and perceptual strategies for exploring it, as well as the difficulties involved in measuring its impact on the perceiving subject, have given rise to three main areas of research focused on both natural and urban landscape assessment:

- Landscape assessment studies focused on the “objective” attributes of the landscape that can be measured directly, usually by expert observers (Prato, 2000; Otero et al., 2007; Vizzari, 2011; Schirpke et al., 2013).
- Landscape preference studies focused on the perceptions of everyday users of the landscape (i.e., non-expert individuals) and concerns about the “nature” of those users' assessments (Hull and Stewart, 1995; Yamashita, 2002; Roth, 2006; Hunziker et al., 2008).
- Studies on the landscape's restorative qualities, focused on the potential impact of interactions between landscape and users (Milligan and Bingley, 2007; Lee et al., 2009; Korpela, 2013; Pazhouhanfar and Kamal, 2014; Finlay et al., 2015).

The aforementioned research areas are, in fact, steps in a process that commences when a subject first comes into contact with a context that can be perceived; a context that, according to some objective characteristics, attracts the attention of the perceiving subject. Because of previous history and expectations, the subject may experience a series of emotions of varying complexity that will shape what is known as the aesthetic experience. Such an experience may sometimes lead to restorative effects in terms of physical and psychological health.

In this sense, for Cuthbert (2006, p. 174), an aesthetically pleasurable experience is one that provides “*pleasurable sensory experiences, pleasing perceptual structure and pleasurable symbolic associations*.” This definition offers us a useful guide for describing the various levels of aesthetic perception involved in the appreciation of an urban space, which could be compared to the appreciation of a work of art, since, according to Fenner (2003): it implies sensorial perception, cognition and meaning. From this perspective, as outlined above, different formal aspects of a specific setting, such as consistency of building styles, colors, and materials, etc., may evoke the visual interest of the perceiver and, together with perceiver's previous experiences in either this or similar settings, may shape the aesthetic experience. In our study, the focus of interest will not be the perceptual processes of the aesthetic experience, but rather the consequences of the experience in terms of restoration.

Environmental psychologists and other scholars and practitioners have been interested in how natural environments contribute to human health and well-being for almost four decades now; although the origins of this approach date back to the last third of the nineteenth century and the works of Frederick Law Olmsted (Twombly, 2010). Research into this topic has generally been based on two different yet equally well-known frameworks: attention restoration theory (ART) developed by Kaplan and Kaplan (1989) and stress recovery theory (SRT), postulated by Roger Ulrich (Ulrich, 1981, 1993; Ulrich et al., 1991).

ART states that natural environments can restore the cognitive resources that people use in their daily performance (work, studies, responsibilities, etc.), as long as they are experienced as psychologically distant from daily context (*being away)*, have a rich, complex, and well-organized content (*extent*), are aesthetic and interesting (*fascination*), and fit their needs and inclinations (*compatibility*). According to this theory, the involuntary attention triggered by natural scenes is responsible for the recovery of voluntary attention and the reduction of the irritability and frustration that stem from attentional fatigue.

For its part, Ulrich's theory postulates that the stress response elicited by some life events, despite its adaptative value, is followed by the consumption of psychological energy and the emergence of a negative emotional state. Thus, a positive affective response to open natural environments will allow the individual to recover from fatigue and its negative emotional outcomes. According to SRT, the main environmental features underlying this emotional reaction are the number of natural elements, the openness, depth, and moderate complexity of the setting and the absence of threats and diversionary demands.

Therefore, the two frameworks give a different degree of prominence to the cognitive and emotional processes and describe the restorative experience in different ways. Nonetheless, both can be understood on the basis of Simmel's classic proposal (1984), which defines the urban environment in terms of an overload of stimuli that leads to saturation, a decrease in social interactions and an undesirable stress response among citizens. In this sense, both agree on the fact that modern life challenges human resources and may lead to a psychological state characterized by low task-performance and negative emotional outcomes (Bratman et al., 2012). These very influential contributions have inspired a substantial body of research, and as a result, a large amount of supporting evidence has been gathered. Research has explored the cognitive and the emotional effects of restorative experiences indistinctly, showing an inherent integration of the two frameworks. Evidence of restorative effects has been found in laboratory (van den Berg et al., 2003; Berto, 2005), field (Hartig et al., 2003; Gatersleben and Andrews, 2013; Tyrväinen et al., 2014), and survey-based studies (Korpela et al., 2010).

### Two possible biases

For decades, an important part of the discourse of and the research conducted in the social sciences has considered cities as settings which could give rise to psychological health problems and social disruption phenomena through social, economic, environmental and spatial factors (Milgram, 1970; Marsella, 1998; Nelson et al., 1998). This negative view of the city may have influenced the study of the psychology of restoration as well. In fact, in the paradigmatic works of Ulrich and the Kaplans we find direct and indirect allusions to the marked contrast between natural and urban environments in terms of *restorative potential*. Both ART and SRT present nature as “healing” and describe cities, or life in cities, as the opposite. The urban environment is consequently seen as more stressful and less attractive than nature, and in some way responsible for the negative effects that then require contact with Nature in order to be redressed.

In this sense, many studies in the last two decades have compared natural environments to urban environments characterized by a high presence of noise, pollution, traffic congestion and, in most cases, little aesthetic value (see for example (Berman et al., 2008; Park et al., 2010; Takayama et al., 2014)). In the words of Karmanov and Hamel (2008) “the urban environments of earlier studies seem often to have been chosen so as to emphasize the difference in restorative potential between nature and city. Not surprisingly, such urban environments were found to have little or no restorative potential” (p. 122). Other authors seem to agree with this analysis (Fornara and Troffa, 2009; Korpela et al., 2010; Fornara, 2011). Moreover, a recent publication pointed out another bias that may have affected research in this area. Staats et al. (2016) claim that when selecting natural environments, studies have chosen ones with recreational purposes, while urban environments, usually streets, were places for transport. Given this possible place selection bias, some of the knowledge gathered to date may somehow be partial and inaccurate.

As a result of the biases described above, previous research may have developed a distorted or misapprehended image of the city's restorative potential, even perhaps contributing to maintain the Manichean urban-nature dichotomy. If so, additional research is required to overcome such limitations, in line with a very recent book on positive environmental psychology that outlines the positive value of urban environments (Corral et al., 2014). This piece of research aims to do just that.

### The emergence of a new question

If the situation is indeed as described in the previous section, new research is required, and this in fact coincides with recent results reported in this area. Several studies have pointed out that not every piece of nature is equally restorative. Natural environments have been found to be more restorative when they offer more prospect and less refuge views (Gatersleben and Andrews, 2013), contain more mystery (Szolosi et al., 2014), are not scary (Milligan and Bingley, 2007), are more fascinating (Berto et al., 2010), and are less “wild” or even less natural (Martens et al., 2011). In relation to this last observation, one might also add that human use of many natural environments, such as deserts, jungles, and mountains, may prove itself not only not restorative but actually dangerous, risky, and harmful. With this in mind, if nature contains different levels of restorative potential, then urban places could be expected to do so also. Furthermore, even in the event of nature being always more restorative than urban environments, this does not necessarily mean that urban places can never be restorative. Theoretically speaking at least, some urban scenes could meet, to some extent, the criteria of restorative places and may therefore be restorative too. One experimental study supports this idea (Karmanov and Hamel, 2008): subjects who watched a 10-min video of a natural landscape reported a significant decrease in three affective variables (anger, tension, and depression), whereas those who watched a video of an urban landscape reported a decrease in just two (anger and tension). So far, these results are compatible with the idea that urban places can be restorative, although, as stated earlier, maybe not to the same degree as natural ones. Taken together, the results of the studies reviewed here suggest that a *restorative environment*, or in general terms a *positive environment* (Corral et al., 2014), may be either humanized nature or naturalized city. In this sense, two areas of future research can be identified: the patterns of humanization and management that make nature more *restorative* and *positive* and the ones that do the same in the urban context.

Additional support for the claim that urban settings might be restorative may come from research about *perceived restorativeness*. Galindo and Hidalgo (2005) published a study in which three kinds of urban environments (cultural/historical, recreational, and panoramic) were perceived as quite restorative by a group of citizens. Using these same categories, subsequent studies (Fornara and Troffa, 2009; Fornara, 2011) found that historical and panoramic urban settings had a similar restorative value to urban green parks. Environmental preference is closely related to actual restoration (Kaplan and Kaplan, 1989), so if people perceive some urban settings as restorative, this may indeed render them restorative. Even if this assumption is accurate, in our view it is necessary to avoid the tautological simplification of considering that “restorative is what is perceived as restorative.” The challenge is to further understand the specific qualities of the urban landscape that might improve the psychological state of citizens.

In this sense, according to a meta-analysis by Stamps (2004), Kaplan and Kaplan's preference matrix offers no clear and conclusive results. As van der Jagt et al. (2014) state, this proposal is based on an evolutionary point of view: aesthetic preferences would have been shaped by survival opportunities, helping humans to make adaptative habitat decisions. This goes along the lines of other evolutionary explanations for cross-cultural consistencies in landscape preferences, both in terms of landscape configuration and composition (Parsons and Daniel, 2002).

However, we could also argue that phylogenetic factors are not the only ones to influence landscape preference. After millions of years of evolution, human beings are something more than the result of having interacted with key elements for survival. In this sense, we should explore not only what every member of the species has in common, but also what belongs to each social group or even to each individual as a result of the relationship established with the environment in a given spatio-temporal context.

Apart from its theoretical interest, research on urban restoration may have a valuable application within health and urban policies. The number of people living in cities all over the world is constantly increasing, as are stress-related problems (Fuller et al., 2007; van den Berg et al., 2007). A recently published paper claims that restorative experiences in urban settings are of particular interest in this context (Staats et al., 2016). Frequent access to nature for citizens may be difficult due to economic, social, and geographical reasons, and restorative urban places (since they are everyday settings, which are easier and cheaper to visit) may therefore be highly beneficial (Subiza-Pérez et al., 2017). Applying the Restorative Environment approach to cities may be an effective way of ameliorating urban life and contributing to citizens' health and well-being.

In this study, we use the field studies method conducted in other works (Park et al., 2010; Roe and Aspinall, 2011; Tyrväinen et al., 2014) to assess the restorative capacity of urban settings. Although there is evidence supporting the idea that public urban parks and university campuses are restorative (Butryn and Furst, 2003; Hansmann et al., 2007; Berman et al., 2008; Peschardt and Stigsdotter, 2013; Weng and Chiang, 2014), these are among the “greenest” urban settings, and our intention is to focus on other kind of urban places where the built environment is the predominant factor.

As stated above, the research question that motivated this study was whether open urban places can be restorative for their users. Thus, we chose two public squares or “plazas” as our experimental settings, in an attempt to overcome the biases described in the previous section. Two hypotheses were established:

- **H**_1_ Participants spending some rest time in public squares will improve their psychological state in both attentional and affective terms therefore showing that public urban squares can be restorative.
- **H**_2_ Urban squares characterized by a greater presence of natural elements, extent and mystery will prove themselves more restorative than squares with lower levels of these variables.

The reason for choosing squares as study settings lies in the sociopetal nature of this kind of urban element. Paul Zucker, in his seminal work “*Town and Square. From the Agora to the Village Green* (1959)” defined the square as the tri-dimensional space formed by the ground, the façades of the surrounding buildings and the sky. Modern publications briefly present them as open spaces surrounded by buildings (Moughtin and Mertens, 2003). The public squares selected for this study have a strong symbolic and institutional value since a government building (in the first square) and a church (in the second) “dominate” the landscape—to use Zucker's terminology. Morphologically speaking, they belong to two different categories of squares, the first being “wide” and the second one “deep,” according to Sitte's typology (cited in, Moughtin and Mertens, 2003). What they both have in common are the possibilities they offer to the urban perceiver, such as increased visual perspective and diversity of uses beyond urban transit. Therefore, these squares are not mere passing places, but rather enclaves that encourage appropriation.

## Materials and methods

### Sample

Forty-six students from the University of Basque Country (35 women, 11 men; mean age 22.15 years) participated in this study. All were students from the Psychology Faculty and had worked for 3.70 (*SD* = 1.62) and 19.91 (*SD* = 9.95) hours respectively the day and week immediately prior to the experiment. Thus, they were expected to show some attentional fatigue and emotional distress due to their daily university activities (attendance at lectures, group-work, and individual study).

### Description of the public squares

**Figure 1:**
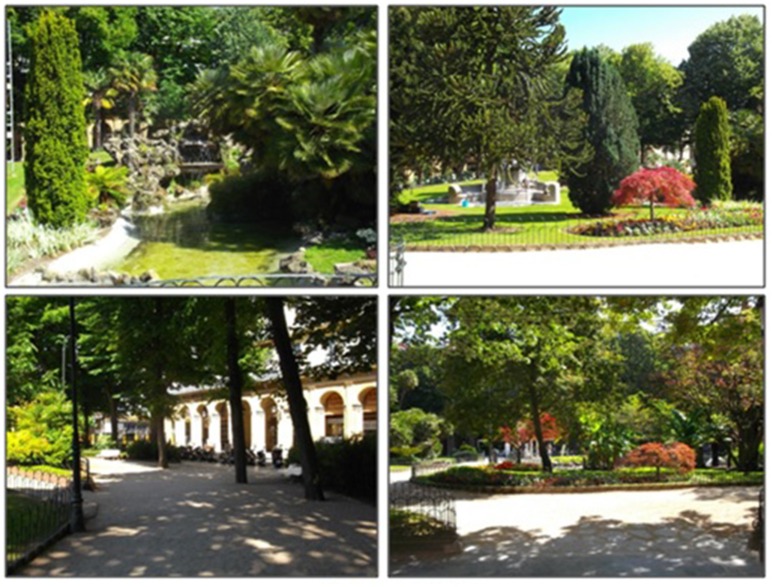
Pictures of place 1.

**Figure 2:**
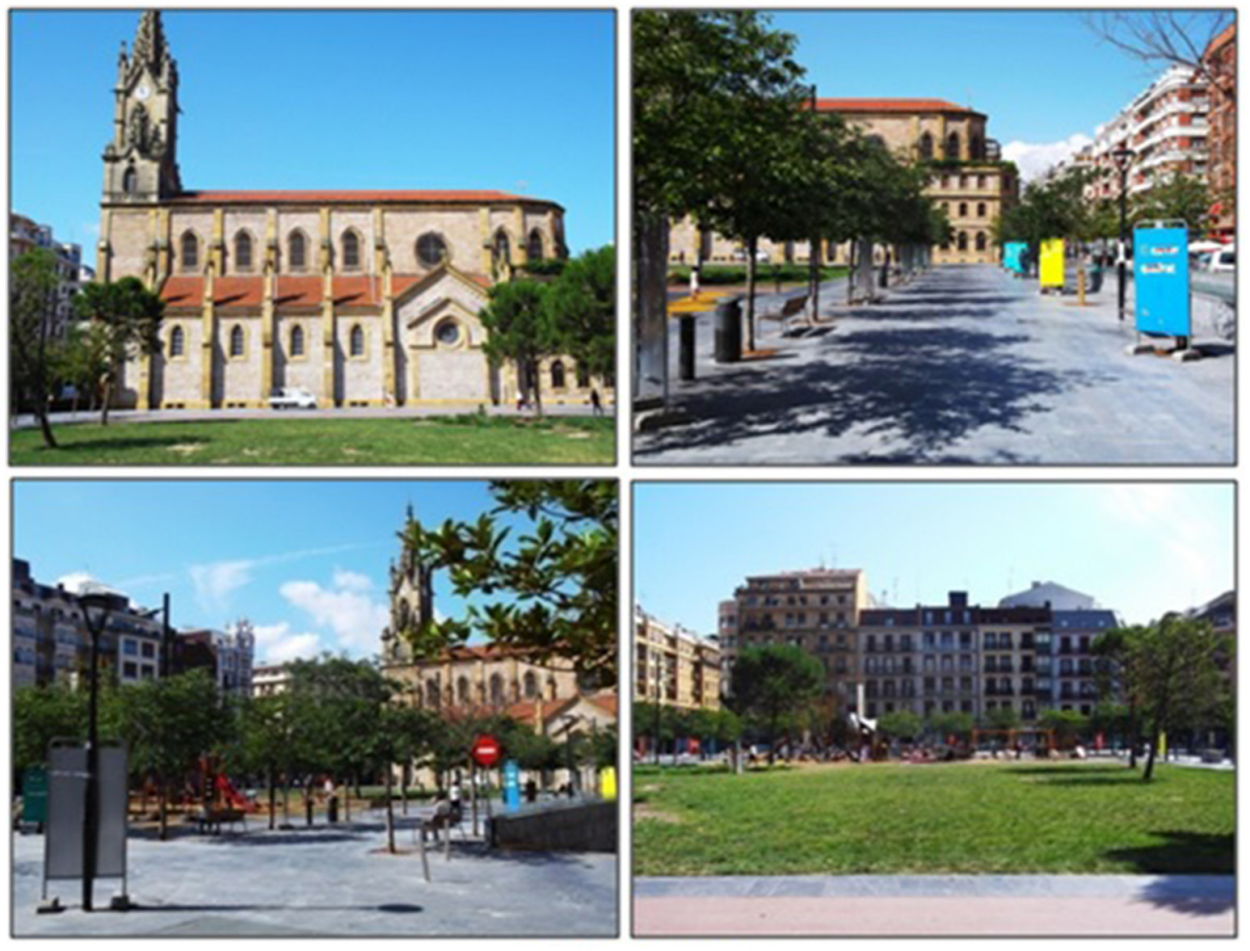
Pictures of place 2.

As stated in the Introduction, the authors were interested in selecting a specific kind of urban environment: the urban “plaza” or square. Thus, two squares were selected: Gipuzkoa Square (public square 1) and Katalunia Square (public square 2), which are representative of the city center and another important neighborhood in the city and are well known by the vast majority of citizens. Figures 1, 2 are photographs of the selected places.

In order to conduct an objective assessment of the squares to be used as experimental settings, a short evaluation sheet was designed by the authors (see Appendix 1). This instrument comprised three different sections focused on the presence of natural elements (i.e., grass, trees, and water), the architectural variation of the surrounding buildings and a series of psycho-environmental variables, including, among others, coherence and mystery. This instrument tried to capture the most important psycho-environmental features related to landscape preference, landscape aesthetics, ART and SRT. The public squares were evaluated by three psychology undergraduates^1^ who collaborated in the research tasks. All were specifically trained in the use of the instrument by the authors. Evaluation results are shown in Table 1.

**Table 1:** Results of the objective environmental evaluation of the two public squares (raters = 3).

	**Public square 1**	**Public square 2**
Natural elements: density [0–21]	10.67 (2.08)	4 (1)
Natural elements: diversity [0–15]	8.67 (0.58)	4.33 (0,58)
Architectural variation [0–16]	1.67 (1.53)	4.67 (1.53)
Psycho-environmental indexes:		
Orientation [0–4]	3 (1)	5 (0)
Coherence [0–4]	3.44 (1.35)	4.33 (0.58)
Enclosure [0–5]	3.56 (0.51)	0.44 (0.51)
Imageability [0–5]	4.22 (0.69)	4.11 (0.84)
Prospect [0–5]	1.33 (0.58)	4.33 (0.58)
Mystery [0–5]	4.33 (0.58)	1 (1.73)
Singularity [0–5]	4.67 (0.58)	3 (1)
Identity [0–5]	5 (0)	3.33 (1.16)
Uniqueness [0–5]	5 (0)	3 (1)
Exploration [0–5]	3.22 (0.51)	2.67 (0.58)
Tranquility [0–5]	2.33 (1.53)	2.67 (1.53)

*The table shows the mean score and standard deviation for each environmental variable assessed by the raters. Greater ratings indicate a higher presence of these environmental features in the setting. Numbers inside square brackets define the range of possible scores for each variable*.

Given the similar place category (square) and the day and hour chosen for the experiment, both public squares were found to have a comparable atmosphere. Since they are squares, they are specially designed to be recreational areas for the city inhabitants and are fully integrated, both physically and symbolically, into the urban dynamics. Because of this, they are well-maintained and equipped. However, a low level of environmental disturbance from traffic and construction work was also perceived. The social landscape is characterized by two main activities. Firstly, a large number of commuters, i.e., people passing through the square on their way to somewhere else. Secondly, a certain number of people spending time in the square, generally chatting with others, contemplating the place while sitting on a bench, looking after children or people with special needs, or simply enjoying a short rest. Public squares 1 and 2 also have some built elements that contribute to the singularity, beauty, and interest of each one of them: works of art and singular constructions (public square 1) and a church, bars, shops, and a children's playground (public square 2).

Despite the similarities described above, however, the two places were selected because of their marked differences in other attributes. Perhaps the most noteworthy contrast lies in the number of green elements. While public square 1 has a large number and variety of perennial trees, plants and grass, public square 2 has much less greenery, just a few deciduous trees with no leaves at the time of the experiment and one small patch of grass. Public square 1 is rich in other natural elements also, since it has a pond with swans and little waterfall.

Public squares 1 and 2 are also dissimilar as regards some of the psycho-environmental factors described in previous literature (Ulrich, 1981, 1993; Kaplan and Kaplan, 1989). Public square 1 has a fair degree of mystery and enclosure while Public square 2 has none of these features. Finally, although public square 2 is more legible, open and with less diversity of elements, the authors believe that, due to its singularity and greenness, public square 1 is more likely to generate a more vivid image and memory.

Consequently, the second study hypothesis is that public square 1, having more greenery, mystery and extent, as well as greater aesthetic potential, will be more restorative than public square 2.

### Instruments

We designed a *Brief General Data Questionnaire*, which included some demographic information (age, gender, and years of residence in the city). Additionally, subjects were asked to report the total number of hours worked on the day of the experiment and since the start of the week (from Monday). The word “work” refers to hours of cognitive performance and voluntary attention activity. It includes attendance at lectures, studying, voluntary service and paid work.

Following Bringslimark et al. (2009), we included instruments used in previous studies in order to move toward a standard measurement kit which will facilitate the comparison between studies and the gathering of results. Examples of the use of these instruments are the works by Bodin and Hartig (2003); Lethbridge et al. (2005); Park et al. (2010); Tsunetsugu et al. (2013), and van den Berg et al. (2003).

Cognitive performance was assessed using the *Symbol Digit Modalities Test* (SDMT), in which subjects are asked to pair specific numbers to symbols in accordance with the given test key at 90-s intervals. The score (120 maximum) is calculated by subtracting the number of errors from the total number of answers. In order to avoid learning effects, we used two parallel versions designed previously (Hinton-Bayre et al., 1997; Hinton-Bayre and Geffen, 2005).

Affective state was measured using the Short Spanish Version of the *Profile of Mood States* (POMS) adapted by Andrade et al. (2013). In this instrument, subjects are asked to rate 25 adjectives expressing affective states on five-point Likert-type scale from 0 to 4 (0 = not at all, 4 = extremely). The items are grouped into five dimensions: tension-anxiety, depression-dejection, anger-hostility, fatigue and vigor. We also used the *Overall Happiness Scale (OHS)* and the *Overall Stress Scale (OSS)*. In both measures, subjects are asked to rate their total happiness and perceived level of stress at the moment of answering, with possible scores from 1 to 100.

Participants also completed the *Perceived Restorativeness Scale* (PRS) (Hartig et al., 1997). This instrument is a widely used 16-item scale that comprises the four main components of Restorative Environments according to ART: *being away, extent (coherence), fascination*, and *compatibility*. Here, the Spanish adaptation (Hidalgo and Hernández, 2001) was used in conjunction with a 6-point Likert-type scale.

For all the instruments used in the study (SDMT, POMS, OHS, OSS, and PRS), higher ratings indicate a larger presence of the variable for the subject in each data collection moment.

### Procedure

Once they had been contacted and informed of the nature of the study, interested students stated their time availability. Using this information, groups of between 3 and 7 participants (X = 4.18; *SD* = 1.54) were formed and randomly assigned to one of the experimental settings on a specific day, with 21 subjects being assigned to public square 1 and 25 to public square 2. Experimental sessions took place between October 14th and November 6th and lasted around an hour and a half, from 15.30 to 17.00.

**Figure 3:**
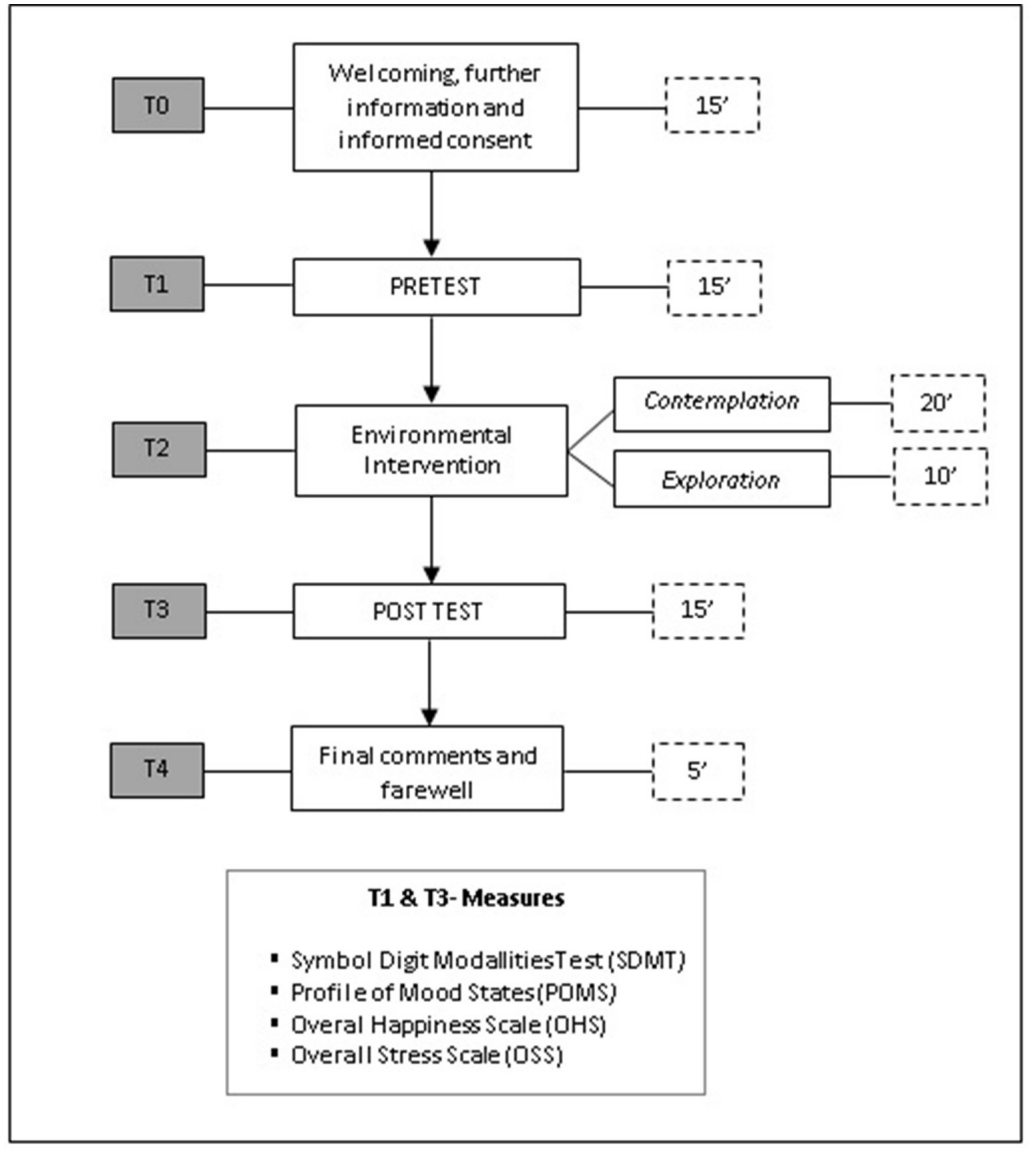
Schema of the experimental procedure designed for this study. Minutes are the unit of time used for the description of each part of the procedure.

Subjects were asked to meet up at a street close to the experimental setting, where they were provided with further information about the activity and research project and gave their informed consent. Before going to the setting, they completed the general data questionnaire and the pretest. After completing the pretest, they were taken to the setting in a <2 min walk and asked to complete the *PRS* before entering the square. They did so in an adjacent street that allowed for visual contemplation of the setting. The environmental intervention designed for this study was inspired by previous research (Park et al., 2010; Takayama et al., 2014; Tyrväinen et al., 2014), and was 30 min long, divided in two phases; static contemplation (20′) and exploration (10′). This environmental intervention design enables two levels of immersion in the landscape: contemplative and explorative. It has been argued that restoration is only achievable if individuals are able to immerse themselves in the environment and feel that they are part of it (San-Juan, 2013). The authors believe that this outcome is easier to achieve in this way than through laboratory or merely contemplative activities. Moreover, the authors were interested in using a similar design to that used in previous research in order to facilitate homogenization and enable comparisons between studies. Thus, participants were first instructed to sit on a bench for 20 min and to contemplate their surroundings, avoiding social interaction, the use of technological devices and the consumption of alcohol or tobacco. In the following exploration phase, they were asked to explore and walk around the square, while still subject to the same restrictions (no social interaction, technological devices, drinking, or smoking). The most usual activities during this phase were walking around, sitting on other benches and contemplating the place from different viewpoints. Finally, participants completed the posttest and, after being thanked, left. A representation of the experimental procedure is showed in Figure 3.

### Data analysis

**Figure 4:**
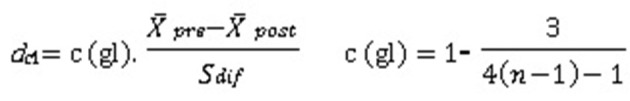
Formula used to estimate the effective size. c(gl) is a bias-correction factor for sample's size.

In accordance with the aims and purposes of the present study, we compared pretest and posttest scores for the cognitive and affective variables included in order to detect any significant differences. As the pretest and posttest data failed to meet normal distribution assumption in most cases, the authors decided to use non-parametric analyses. After detecting statistically significant differences, effect size was calculated using d_c−1_ (Botella and Sánchez-Meca, 2015). This is a specific effect size index suitable for designs with repeated measures (see Figure 4). Additionally, an inter-subject comparison of the PRS scores for the experimental settings was run using analysis of variance (ANOVA), as these data did meet the normality assumption.

## Results

### Analysis regarding H_1_

#### Initial psychological state

Before focusing on any possible restoration achieved by participants, their psychological state at the start of the experiment was analyzed. According to pretest measures, even though subjects had worked an average of almost 4 h on the day of the experiment and 20 since the beginning of the week, their attentional fatigue and emotional distress levels could be described as low. *SDMT* scores revealed a low error rate and a good level of general performance. Participants' scores were low to very low for *tension-anxiety, depression-dejection, anger-hostility*, and *fatigue*. Finally, they reported a medium-low level of *stress* and a medium-high level of *happiness*.

An initial Mann Whitney U test was carried out to detect whether there were any significant differences between groups as regards the number of hours worked and their psychological condition at the beginning of the experiment. No differences were found. The scores for each variable and group are shown in Table 2.

**Table 2:** Initial scores for subjects in public squares 1 and 2.

**Measure**	**Public square 1**	**Public square 2**	**U**	***p***
Hours worked that day	3.38 (1.88)	3.96 (1.36)	187	0.089
Hours worked that week	19.91 (10.59)	19.92 (9.61)	262	0.991
*SDMT*—Errors	1.33 (1.39)	1.04 (1.65)	217	0.287
*SDMT*—General Score [0–120]	60.86 (7.34)	57.08 (8.92)	200.50	0.171
*Tension-Anxiety* [0–4]	1.01 (0.86)	1.06 (0.83)	246	0.714
*Depression-Dejection* [0–4]	0.2 (0.38)	0.44 (0.52)	179.50	0.051
*Anger-Hostility* [0–4]	0.42 (0.64)	0.46 (0.67)	247	0.718
*Fatigue* [0–4]	0.99 (0.86)	1.23 (1.02)	227	0.431
*Vigor* [0–4]	2.04 (0.78)	1.63 (1.06)	195	0.136
*Stress* [0–100]	37.05 (21.94)	41.60 (24.18)	224.50	0.398
*Happiness* [0–100]	69.81 (16.96)	59.40 (18.22)	185	0.085

*Mean, standard deviation of public square 1 and public square 2 for pretest scores, Mann-Witney U value and p value are given for each of the above variables. Numbers inside square brackets define the range of possible scores for each variable*.

#### Restoration obtained in public square 1

The Wilcoxon Signed Rank Test revealed significant differences between pretest and posttest scores for *SDMT-*Errors, *SDMT*-General score, *tension-anxiety, anger-hostility, fatigue, vigor, stress*, and *happiness*. The effect sizes of these differences were low to medium (d_c1_ = 0.33–0.59) except for *Stress*, which was high (d_c1_ = 0.85). Differences in *depression-dejection* were not significant. For further information, see Table 3.

**Table 3:** Pretest-Posttest differences for public square 1.

**Measure**	**Pretest**	**Posttest**	**Z (W)**	***p***	***d*_*c*1_**
*SDMT*–Errors^*^	1.33 (1.39)	0.67 (0.91)	−2.170	0.03	0.33
*SDMT*–General score^*^	60.86 (7.34)	64.67 (8.52)	−2.468	0.014	0.59
Tension-Anxiety^**^	1.01 (0.86)	0.35 (0.42)	−3.370	0.001	0.50
*Depression-Dejection*	0.2 (0.38)	0.19 (0.28)	−0.081	0.935	–
Anger-Hostility^**^	0.42 (0.64)	0.13 (0.27)	−2.697	0.007	0.59
Fatigue^*^	0.99 (0.86)	0.74 (0.82)	−2.272	0.023	0.44
*Vigor*^2^^*^	2.04 (0.78)	1.71 (0.62)	−2.490	0.013	0.58
Stress^**^	37.05 (21.94)	23.38 (19.03)	−3.257	0.001	0.85
*Happiness* ^**^	69.81 (16.96)	76.48 (15.06)	−2.810	0.005	0.59

*Mean, standard deviation of public square 1 for pretest and posttest scores, W Wilcoxon statistic value and p value are given for each of the above variables*.

* p-value < 0.05 and

** *p-value < 0.01*.

#### Restoration obtained in public square 2

The Wilcoxon Signed Rank Test revealed significant differences between pretest and posttest scores for *SDMT*-General score, *tension-anxiety, anger-hostility, fatigue, stress*, and *happiness*. The effect sizes of these differences were medium for *anger-hostility, fatigue* and *happiness* (d_c1_ = 0.53–0.63), high for *SDMT*—General score and *tension-anxiety* (d_c1_ = 1.11 and 1.05) and very high for *stress* (d_c1_ = 1.60). Differences in the *depression-dejection* and *vigor* were not significant. Further information is provided in Table 4.

**Table 4:** Pretest-Posttest differences for public square 2.

**Measure**	**Pretest**	**Posttest**	**Z(W)**	***p***	***d*_*c*1_**
*SDMT*—Errors	1.04 (1.65)	0.60 (0.82)	−0.898	0.369	–
*SDMT*—General score^***^	57.08 (8.92)	62.08 (9.45)	−3.954	0.0001	1.11
Tension-Anxiety^***^	1.06 (0.83)	0.42 (0.56)	−3.962	0.0001	1.05
*Depression-Dejection*	0.44 (0.52)	0.37 (0.46)	−1.044	0.296	–
Anger-Hostility^**^	0.46 (0.68)	0.17 (0.39)	−2.821	0.005	0.53
Fatigue^**^	1.23 (1.02)	0.79 (0.63)	−2.738	0.006	0.63
*Vigor*	1.63 (1.06)	1.50 (0.87)	−0.540	0.589	–
Stress^***^	41.60 (24.18)	17.60 (15.62)	−4.302	0.0001	1.60
*Happiness* ^**^	59.40 (18.22)	69.40 (14.24)	−2.924	0.003	0.62

*Mean, standard deviation of public square 2 for pretest and posttest scores, W Wilcoxon statistic value and p-value are given for each of the above variables*.

** p-value < 0.01 and

*** *p-value < 0.001*.

### Analysis regarding H_2_

As it is shown in Table 5 *PRS* scores for each place were compared using an ANOVA. PRS overall score in public square 1 was greater than in public square 2. It also scored higher than public square 2 in *being away* and *fascination*.

**Table 5:** PRS Scores by public square and ANOVA results.

**Measure**	**Public square 1**	**Public square 2**	***F*_(1, 44)_**	***p***
PRS—Overall score^**^	2.53 (0.59)	2.07 (0.59)	7.41	0.009
Being away^*^	2.69 (0.78)	2.18 (0.88)	4.14	0.048
Coherence	3.11 (0.61)	3.02 (0.89)	0.14	0.706
Extent	2.11 (0.74)	1.89 (0.69)	1.05	0.311
Fascination^***^	2.17 (0.74)	1.30 (0.60)	19.13	0.0001
Compatibility	2.65 (0.80)	2.20 (0.85)	3.40	0.072

*Mean and standadr deviation of PRS scores by public square. F statistic and p-value are given for each of the above variables*.

* p value < 0.05;

** p-value < 0.01; and

*** *p-value < 0.001*.

**Figure 5:**
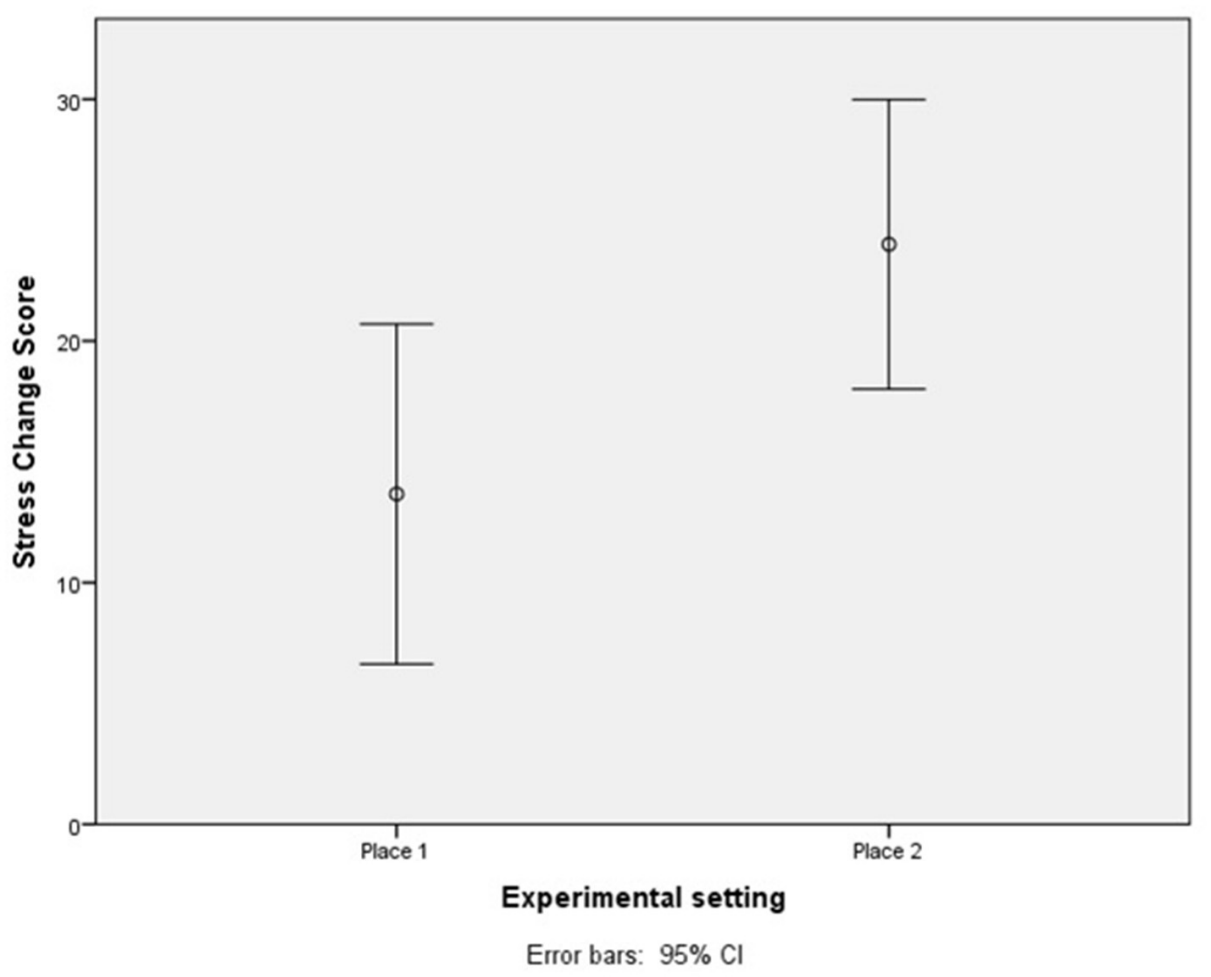
Stress change scores per experimental setting. Higher rates indicate higher stress reduction in the setting.

A quick look at Tables 3, 4 reveals some dissimilarities in the effect size of the pretest-posttest differences for some variables (*SDMT-GS, T-A*, and *stress*). This might indicate that participants in public square 2 experienced a greater restoration of such variables, suggesting that the second setting may be more restorative than the first one. To further explore this possibility, the restoration obtained per group was operationalized as the change between pretest and posttest scores. Those scores were then compared to check if there were significant differences between places. Once again, due to a non-normal distribution of data, a non-parametric test was used (the Mann Whitney U test). The results of this analysis revealed statistically significant differences only in the change reported in *stress* (*U* = 153, *p* = 0.015, d_c1_ = 0.70). For further information about this analysis, see Table 6. For a graphic depiction of the stress change scores, see Figure 5.

**Table 6:** Change scores by public square for selected variables.

**Measure**	**Change in public square 1**	**Change in public square 2**	**Z(U)**	***p***	***d*_*c*1_**
*SDMT*—General score	3.81 (6.19)	5 (4.73)	−0.265	0.791	–
*Tension-Anxiety*	0.66 (0.69)	0.65 (0.60)	−0.212	0.832	–
Stress^*^	13.67 (15.45)	24 (14.51)	−2.434	0.015	0.70

*Mean, standard deviation of change scores for SDMT general score, tension-anxiety and stress. Mann Witney U Z value and p-value*.

* *p-value < 0.05*.

## Discussion

The main aim of this study was to test the *restorative potential* of public squares in order to provide an, at least initial, answer to the research question (Are public open places restorative?). This is an idea that has been proposed previously by other authors (Galindo and Hidalgo, 2005; Karmanov and Hamel, 2008; Fornara and Troffa, 2009; Fornara, 2011). The first hypothesis (**H**_1_) proposed that public urban squares are restorative. The results obtained support this hypothesis, since participants reported a better psychological state after spending some time in an urban square. Visitors to both places had better cognitive performance, reduced negative affect variables (tension-anxiety, anger-hostility, fatigue, and stress) and reported an increase in happiness after spending 30 min in the square. These results may provide information about the existence of the restoration process, or a similar one, in urban environments. This set of results is relevant due to the existence of previous research finding no restoration, or even documenting a deterioration of psychological variables, in urban contexts (Berman et al., 2008; Park et al., 2010; Tsunetsugu et al., 2013; Takayama et al., 2014). In this point it is also worthy to recall a study conducted by Roe and Aspinall (2011; Study 2) showing that an urban walk produced a significant increase in hedonic tone and stress reduction in a sample of adults with poor mental health.

The second hypothesis of the study (**H**_2_) stated that due to some of its features (greenness, mystery, and extent), and according to the classical premises of restoration theory, public square 1 would elicit higher restoration rates than public square 2. Participants' ratings of the Places were found to be congruent, with public square 1 scoring higher for *perceived restorativeness* (overall score), *being away*, and *fascination*. Results showed that participants achieved restoration in most of the variables studied, regardless of the public square to which they were assigned, and a comparison of the restoration rates revealed that visitors to public square 2 achieved a significantly higher level of stress reduction. Since no other differences between the squares were found, it could be concluded that their restorative potential is similar. This finding does not allow **H**_2_ to be accepted and poses a number of questions since it is not consistent with some of the most widely-accepted premises of restoration theory. In the following paragraphs, we will try to reflect on this and offer various possible explanations.

From a theoretical point of view, the results obtained may be seen as controversial. ART postulates that greenness and mystery are two of the key features of restorative places. Following such postulates, public square 1, which has a higher level of mystery and many more trees, flowers, grass and natural elements than public square 2, should have been more restorative. Existing evidence linking greenness to healthiness (Smardon, 1988; Lee and Maheswaran, 2010; Pearson and Craig, 2014) would also seem to suggest this outcome. We must therefore turn to other approaches in order to explain the results obtained here. White et al. (2010) found that water, an important natural feature for human survival and evolution, and hence related to restoration, functioned differentially in natural and urban settings. Indeed, the more water in the natural pictures, the more perceived restorativenness (PR), whereas in urban pictures, only the presence of water was significant and its increase did not generate more PR. Two ideas emerge from this finding: firstly, that the presence of a specific element, such as water or trees, for example, in urban settings *(conceptual presence)* might be more influential than its density or the proportion of the place that it takes up; and secondly, that elements that are thought to be important in the context of natural restoration may have a lesser significance in urban settings.

SRT states that, to be restorative, places should be open and have enough prospect. In this sense, since public square 1 is more enclosed, it might be less restorative than public square 2. Studies have found that enclosure, defined as lack of prospect and availability of hiding places could increase perceptions of insecurity (Nasar and Jones, 1997; Stamps, 2005; Foster et al., 2010; Cinar and Cubukcu, 2012). Indeed, the visual and/or locomotive impermeability of green items have been identified as important features related to insecurity in urban contexts (see also Herzog and Chernick, 2000). Therefore, the more enclosed setting (public square 1) may provide a lower level of restoration, in accordance with the postulates of Herzog and Rector (2008)^3^. We are not suggesting that participants experienced insecurity in public square 1, especially given their profile, the time of the experiment and the overall level of safety in the city, but enclosure could have been an obstacle for achieving a certain level of restoration.

Another possible (and probably complementary) explanation may be the previously mentioned information overload framework. One of the main attributes of urban life is the huge amount of information and the plethora of stimuli that surround people and their daily life performance (Milgram, 1970; Johansson et al., 2011; Staats et al., 2016). Classical studies on this topic focused on place-based sources such as crowds, the media, population density, and noise, etc., but in the twenty-first century, digital sources of overwhelming information should also be taken into account (Misra and Stokols, 2012). In a context in which individuals are constantly bombarded by massive amounts of information from their immediate physical and social environments, along with an endless flood of data from the world of technology and internet-based devices, complex, mysterious, rich urban settings full of stimuli may be less restorative. Due to information overload, citizens may well find quiet, open places with a greater prospect and fewer stimuli more relaxing and refreshing than more stimulating ones. From this perspective, public square 1 may have been more psychologically demanding, and therefore less restoring than public square 2. This idea is further supported by the significant decrease in Vigor reported by participants who visited public square 1 (Z = −2.490, *p* = 0.013, d_c1_ = 0_._58). In other words, calm and low-to-medium stimulating settings would be more likely to offer the *soft fascination* needed for restoration. In any case, further research is required using more types of urban settings, with various levels of the aforementioned dimensions, in order to replicate our findings and gather more evidence of the relevance of each dimension in the city.

## Limitations and future research directions

The study has a number of limitations which help us define new lines of enquiry for consolidating the research initiated here. Although this is quite frequent in previous experimental research conducted in this area, the sample group was small and composed exclusively of university students. Secondly, the absence of a control group is a weakness that invites to conduct further research using control groups to increase the internal validity of the study. Additional limitations may be that the sample was not balanced in terms of gender and that working with psychology students might have led to a certain degree of bias because they were in a better position to guess the objectives of the study. Broader and more heterogenic samples of citizens will be required to replicate the results and, as stated previously, more (and more diverse) settings should be used in future studies to consolidate this avenue of research into restorative urban settings. Moreover, in our study, an improvement in psychological measures was detected even when the initial state of the participants was not particularly negative in terms of stress and fatigue. A replication with a fatigued or stressed sample group is therefore required, since the effect size of the restoration processes may be larger in this case. As regards instruments, only psychological measures were used, and physiological ones may be also useful in this context. The devices and technologies that we now use every day, such as smartphones or watches, may prove useful for collecting heart rate data and other measures. Neither the objective nor the subjective assessment of the experimental setting comprised items linked to the soundscape (natural sounds such as animals, water, etc. and urban sounds such as traffic and road works). Future studies may wish to address the contribution of these elements to the restorative experience. This was an exploratory and pilot study, but in spite of these limitations, the authors believe that it is an interesting and promising avenue of research that has substantial applied potential.

In this sense, and following Sörqvist (2016), one of the challenges in environmental psychology is to not take for granted that the built environment is inherently harmful to human well-being, while the natural environment is inherently beneficial. As we stated above, interaction with a virgin, non-humanized, hostile environment may be dissuasive for creating bonds or appropriation processes. On the other hand, as San Juan and Vozmediano (2016) suggest, the city is the place where cultural exchange and socialization processes occur, the physical and symbolic reflection of a community. As indicated by authors such as Jacobs (1961), Alexander (1965), and Gehl (1987), among others, urbanism may be a strategy for developing quality of life, health, solidarity, and democracy, but only if we recognize that urban space is not what remains after locating the buildings; on the contrary, urban design has the potential to create restorative places.

In short, it might be important to remember a very simple fact. ART, SRT, and most of the work they have inspired have been aimed at exploring the effects of nature on psychological health. Thus, it would not be misdirected to think that their approach might require some adaptation—or extension—in order to understand urban restoration. Further research is therefore needed to develop a better understanding of these processes and to collect a broader body of evidence. This brings us back to the idea of the levels of urban naturalization proposed in the Introduction, and we should remember that friendly urban settings may be more affordable, more accessible to more people, and offer a wider variety of possibilities for action and restoration than some natural contexts (wild, virgin, extreme). The study of urban restoration is an important research challenge for both scholars and practitioners, since the applied perspective could provide substantial improvements for the quality of urban life over the coming decades.

## Conclusion

At the beginning of the twentieth century, only 10% of the world's population lived in urban areas. According to recent estimates, this figure will rise to 66% of the population by 2050 (United Nations, 2014), revealing the remarkable success of the urban life. But as the saying goes, too much success can kill you. This study reveals that participants' psychological state improved after spending half an hour in one of the two selected urban squares. These results lead us to conclude that avoiding the collapse of the urban model due to unsustainability cannot be our only aim in relation to the future of our cities. While an adequate management of urban resources, waste and movement flows is necessary, we argue that urban design can also significantly contribute to improving citizens' well-being and quality of life, reducing their stress and restoring their psychological state. Future research could even reflect on how to design aesthetically pleasurable urban landscapes that we could describe as “*emopetal*,” i.e., capable of generating positive emotional experiences.

## Ethics statement

This study was carried out in accordance with the recommendations of “The Research Ethics Committee for Research on Human Beings [Comité de Ética para las de Investigaciones relacionadas con Seres Humanos (CEISH)]” participants. All subjects gave written informed consent in accordance with the Declaration of Helsinki. The protocol was approved by the Research Ethics Committee for Research on Human Beings.

## Author contributions

CS, MS-P, and LV have contributed equally to the design and development of the study here presented, taking an active role in its conceptualization, data collection, statistical analyses and writing of the paper.

### Conflict of interest statement

The authors declare that the research was conducted in the absence of any commercial or financial relationships that could be construed as a potential conflict of interest.

## Footnotes

^1^Although only the independent evaluation results are presented here, the authors also evaluated both settings using the same tool, obtaining similar results to the ones shown here.

^2^Contrary to expectations, the Vigor score in the posttest was lower than in the pretest, indicating a possible decrease in Group 1 participants' energy during the experiment.

^3^Herzog and Rector postulated that coping with danger or fear concerns or situations will disrupt or negatively affect the process of restoration in that it requires direct attention efforts and may generate a negative affect response.
