# Supplementary material for: Restoration and the City: The Role of Public Urban Squares
Source: Front Psychol. 2017 Dec 7;8:2093. doi: 10.3389/fpsyg.2017.02093 (PMC5725966; doi:10.3389/fpsyg.2017.02093)
Supplement: Supplementary file 1 [file DataSheet1.pdf]

Appendix 1. Tool used for the objective assessment of the experimental settings.

Assessed place: ____________
Date: __________
Evaluator: ________________

NATURAL ELEMENTS	DENSITY				DIVERSITY (SPECIES)		
Are there the following green elements in the evaluated space? Please, mark their level of density, diversity and aesthetic potential.	**Absent**	**Low**	**Medium**	**High**	**1-3**	**4-6**	**7+**
Trees	☐	☐	☐	☐	☐	☐	☐
Undergrowth/brushwood	☐	☐	☐	☐	☐	☐	☐
Flowers	☐	☐	☐	☐	☐	☐	☐
Pots or similar	☐	☐	☐	☐	☐	☐	☐
Grass	☐	☐	☐	☐	☐	☐	☐
Lake, pond or similar	☐	☐	☐	☐			
River, stream or similar	☐	☐	☐	☐			

ENCLOSURE						
	**Never (0)**	**Hardly ever (1)**	**Sometimes (2)**	**Often (3)**	**Most times (4)**	**Always (5)**
When in the place, how often can the person be seen by other people placed in the surroundings?	☐	☐	☐	☐	☐	☐
How often can the person visually anticipate any circumstance that could result in danger or rejection?	☐	☐	☐	☐	☐	☐
How often can the person get away in several directions from any circumstance that could result in danger or rejection?	☐	☐	☐	☐	☐	☐

PSYCHO-ENVIRONMENTAL INDEXES					
Orientation	**Very difficult (0)**	**Difficult (1)**	**Neither difficult nor easy (2)**	**Easy (3)**	**Very easy (4)**
Navigating through the place and following the paths is..	☐	☐	☐	☐	☐
Coherence<br><br>*Mark your level of agreement with the following statements.*	**Spatial organization**				
	**Strongly disagree (0)**	**Disagree (1)**	**Neither agree nor disagree (2)**	**Agree (3)**	**Strongly agree (4)**
The space is organized in a coherent manner	☐	☐	☐	☐	☐
Elements here match with each other in a natural way	☐	☐	☐	☐	☐
There is a clear sense of order in this place	☐	☐	☐	☐	☐

Please, mark to what extent do you think that the place presents the following features (0= Not at all, 1=Almost nothing, 2=A little, 3= Some, 4=Quite a lot, 5= A lot).	**0**	**1**	**2**	**3**	**4**	**5**
Prospect<br>Further parts of the place can be seen from the standing point of the observer.	☐	☐	☐	☐	☐	☐
Mystery<br>There are parts or zones of the place that are not strictly observable from the standing point of the observer and that can be intuited behind natural or built covers.	☐	☐	☐	☐	☐	☐
Singularity<br>The place is distinct to its immediate urban environment	☐	☐	☐	☐	☐	☐
Identity<br>The place has an own identity within the city	☐	☐	☐	☐	☐	☐
Uniqueness<br>The place is unique in within the city	☐	☐	☐	☐	☐	☐
Exploration						
The place can be explored in several directions	☐	☐	☐	☐	☐	☐
The place invites to be explored	☐	☐	☐	☐	☐	☐
